# Emerging Role of Extracellular Vesicles in Immune Regulation and Cancer Progression

**DOI:** 10.3390/cancers12123563

**Published:** 2020-11-28

**Authors:** Sonam Mittal, Prachi Gupta, Pradeep Chaluvally-Raghavan, Sunila Pradeep

**Affiliations:** 1 Department of Obstetrics and Gynecology, Medical College of Wisconsin, Milwaukee, WI 53226, USA; smittal@mcw.edu (S.M.); prgupta@mcw.edu (P.G.); pchaluvally@mcw.edu (P.C.-R.); 2 Cancer Center, Medical College of Wisconsin, Milwaukee, WI 53226, USA; 3 Department of Physiology, Medical College of Wisconsin, Milwaukee, WI 53226, USA

**Keywords:** extracellular vesicles, cancer, tumor microenvironment, immune cells, immunotherapy, biomarkers

## Abstract

**Simple Summary:**

Accumulating evidence has reported that extracellular vesicles secreted by different tumor microenvironment cells can interfere with the host immune system. These vesicles transmit the signals in the tumor microenvironment that affect the proliferation, apoptosis, activation, and, metabolism of immune cells such as dendritic cells, T cells, macrophages, and natural killer cells, creating a pro-tumoral environment for tumor progression and survival. In this review, we summarize the recent literature on the function of extracellular vesicles derived from tumor cells and immune cells in regulating the critical processes associated with cancer progression. Besides, we also provide insights on how the extracellular vesicles are employed as diagnostic and prognostic biomarkers and drug carriers in cancer.

**Abstract:**

The development of effective therapies for cancer treatment requires a better understanding of the tumor extracellular environment and a dynamic interaction between tumor cells, the cells of the immune system, and the tumor stroma. Increasing evidence suggests that extracellular vesicles play an important role in this interaction. Extracellular vesicles are nanometer-sized membrane-bound vesicles secreted by various types of cells that facilitate intracellular communication by transferring proteins, various lipids, and nucleic acids, especially miRNAs, between cells. Extracellular vesicles play discrete roles in the immune regulatory functions, such as antigen presentation, and activation or suppression of immune cells. Achieving therapeutic intervention through targeting of extracellular vesicles is a crucial area of research now. Thus, a deeper knowledge of exosome biology and the molecular mechanism of immune regulation is likely to provide significant insight into therapeutic intervention utilizing extracellular vesicles to combat this dreadful disease. This review describes the recent updates on immune regulation by extracellular vesicles in cancer progression and possible use in cancer therapy.

## 1. Introduction

Extracellular vesicles (EVs) represent an extensive family of lipid bilayer-derived vesicles that are known to be released almost ubiquitously by every living cell [1,2]. Among EVs, exosomes function as drivers of intracellular communication. Additionally, in the living system, exosomes act as the “middle man” engaged in the transport of cellular cargo of macromolecules, including proteins, lipids, and nucleic acids [3] The biogenesis of exosomes is a highly dynamic yet regulatory cellular phenomenon. 

Apart from merely acting as vesicles known to dispose of cellular waste, EVs perform distinct roles in many aspects of protein trafficking, extracellular signaling, and immunology [4,5]. The role of each EV varies depending upon their cellular origin. To execute specific functions, such as antigen presentation, immune activation and suppression, immune surveillance, tumor growth, metastasis, and oncogenic transformation, several components are present on the EVs. EVs carry T and B cell receptors, cytokines and cytokine receptors, integrins, lectins, Fas ligand(FasL), miRNAs, sphingolipids, major histocompatibility complex(MHC) class I and II molecules, galectin-9, tumor necrosis factor alpha (TNF-α), tumor growth factor beta (TGF-β), NKG2D ligand, and others [6,7,8]. Tumor-derived extracellular vesicles (TD-EVs) are the product of tumor cells and were first discovered in the extracellular spaces of tumor cell lines [9,10]. The molecular composition of TD-EVs is different from exosomes produced by normal cells but matches with their parent cells. Additionally, the molecular signatures of TD-EVs vary from one type of cancer cell to another [11]. TD-EVs act as a communication system used by tumor cells to deliver signaling molecules to other cells populating the tumor microenvironment (TME). The immunologically active EVs secreted by tumor cells and immune cells can inhibit or promote tumor growth by regulating the antitumor immune response [12,13]. The concentration of EVs is high in plasma or body fluid in cancer patients as compared to normal counterparts [14,15]. Currently, TD-EVs are of special interest in cancer research because of their potential to act as noninvasive tumor biomarkers and to modulate the immune response [2,16]. This review focuses on understanding the role of extracellular vesicles in immune regulation and its therapeutic potential in cancer.

## 2. Impact of Extracellular Vesicles on Different Immune Cells

Exosomes were first discovered as endosomal-derived EVs in the maturing mammalian reticulocyte (immature red blood cell) in 1983 and the term “exosome ”was coined a few years later in 1987 [17,18,19]. B cell-derived exosomes were later described in late 1990s. Subsequently, various studies have also reported the association of exosome-like structure with all types of immune cells, such as B and T lymphocytes, dendritic cells (DCs), natural killer (NK) cells, mast cells, macrophages, and thymocytes. Exosomes derived from immune cells play an important role in regulating the immune response in many events. These vesicles are enriched in proteins with immune-modulating functions viz. MHC-I, MHC-II, costimulatory proteins (CD86), and adhesion proteins (CD11b, CD54/ICAM). EVs derived from antigen-presenting cells (APCs) and other immunocytes [20,21,22,23], which express antigen-presenting molecules (MHC-I and MHC-II) on their surface, present antigen to T cells and activate the immune response [24]. EVs can present antigen to T cells either through direct presentation or cross-presentation. In direct presentation, EVs present MHC–peptide complexes directly to antigen-specific T cells, thereby leading to T cell activation. In cross-presentation, APCs process the antigen carried by EVs and present them to CD8^+^ T cells [25,26].

## 3. Functional Regulation of Immune Cells by Tumor-Derived Extracellular Vesicles

**Figure 1:**
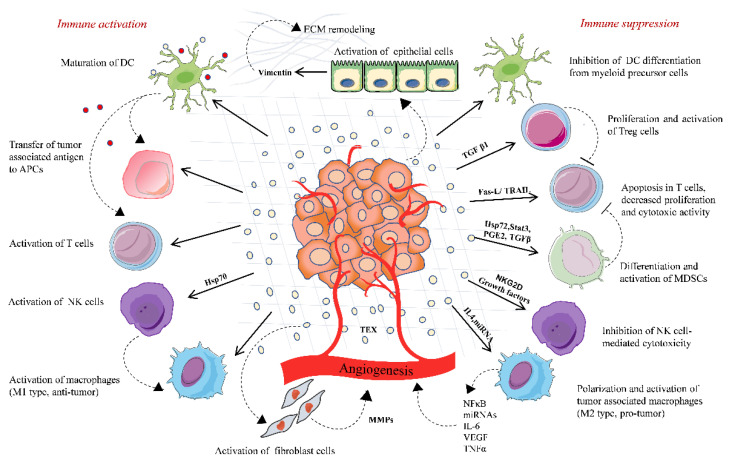
Tumor-derived extracellular vesicles-based immune regulation in the tumor microenvironment. TD-EVs regulate the immune system homeostasis by playing a dual role, either acting as an immune activator or mostly by activating immunosuppressive changes that promote tumor. TD-EVs activate and proliferate Treg cells and MDSCs, which suppress the immune response against the tumor. TD-EVs containing FasL or TRAIL induce the apoptosis of CD8^+^ T cells and suppress the activation of T cells. TD-EVs deliver TAA to DCs and activate T cell-mediated antitumor response. TD-EVs either activate or suppress the NK cells depending upon the type of cargo they carry. Similarly, TD-EVs activate macrophages either toward M1 type (antitumor) or M2 type (protumor). TD-EVs play a significant role in remodeling of the extracellular matrix and angiogenesis, thereby promoting the release of tumor cells in circulation and their invasion into the distant organ.

The process of EV transfer is well regulated at many steps so that the information is transferred to the specific recipient. It is believed that transfer is mediated by the ligand–receptor type of interactions followed by endocytosis. Valadi et al. showed that EVs transfer mRNAs and miRNAs from one cell to another and induce cellular changes in the recipient cells [27]. Al-Nedawi et al. showed EVs-mediated transfer of EGFR from cancer cells to endothelial cells [28]. However, the mechanism of transfer of TD-EVs and their cargo in recipient cells is not yet understood. Increasing evidence reports that EVs play a dual role in regulating immune response either by immune suppression or stimulation due to their complex structure (Figure 1). The immune regulatory functions of TD-EVs are discussed herein the following sections. 

### 3.1. Tumor-Derived Extracellular Vesicles and Suppression of the Antitumor Immune Response

Despite the advancements in immunotherapy, such as immune checkpoint inhibitors (anti-PD-1, anti-PD-L1, and anti-CTLA-4) and CAR-T cell therapies, patients with advanced-stage solid cancer often display inadequate response and resistance to these therapies [29,30,31]. The mechanisms responsible for tumor immune escape and immune suppression in cancer have been extensively studied and it varies from one tumor type to others. Interestingly, EVs secreted by cancer cells have demonstrated a possible impact on tumor cells to evade the antitumor immune response, which is strictly dependent on the type of immunoinhibitory molecules on EVs and the presence of relevant receptors on targeted immune cells [13]. Recent findings have reported several distinct mechanisms harnessed by TD-EVs to escape from immune responses, such as:

**(a)**  Impaired antigen presentation: Tumor cells express antigens called tumor-associated antigen (TAA), which could be either mutated or abnormal proteins with distinct post-translational modifications. TAA and their peptides are presented to the cell surface by MHC I complex are recognized and destroyed by cytotoxic T lymphocytes (CTLs), resulting in cancer cell killing [32]. However, cancer cells can escape from this destruction. This is achieved by downregulating MHC I expression, which may affect the antigen processing machinery and leads to defective antigen presentation. Thus, the cancer cell lacking target antigen/MHC I expression is no longer recognized by CTLs but eventually recognized and destroyed by NK cells according to the missing-self hypothesis [33]. To escape from NK cell-mediated killing, cancer cells may release EVs that affect the NK cell-mediated cytotoxicity by regulating the expression of NK cell-activating NKG2D receptor [34]. NKG2D receptor interacts with its ligands MIC-A and MIC-B (MHC class I chain-related proteins A and B) and UL-16 binding proteins (ULBPs). EVs carrying NKG2D ligands (MIC-A/B and ULBPs) decrease the NKG2D expressions on NK cells and impair NKG2D-mediated NK cell cytotoxicity in acute myeloid leukemia, mesothelioma, prostate, and breast cancer cells [34,35,36].
**(b)**  Inhibition of antigen-presenting cells and cytotoxic T cell- EVs derived from sera of melanoma and head and neck cancer patients inhibited the proliferation of CD8^+^ CTLs. The importance of FasL-, TNF-related apoptosis-inducing ligand(TRAIL-), and PD-L1-containing vesicles in inducing T cell apoptosis has been demonstrated by various scientific groups, including annexin V binding, cytochrome *c* release from mitochondria, loss of the mitochondrial membrane potential, caspase 3-cleavage, and DNA fragmentation [37,38,39]. TD-EVs also targets the PI3k/AKT pathway in activated CD8^+^ T cells by Akt dephosphorylation, which leads to the activation of pro-apoptotic protein Bax and downregulates anti-apoptotic Bcl-2 family members [40]. Moreover, EVs can modulate gene expression profile and function of recipient cells by transferring nucleic acids, especially mRNA and miRNAs. In a study by Muller et al., the EVs from cancer cells induced changes in mRNA expression levels of immune function-related genes in activated T cells. The incubation of TD-EVs with human CD4+ CD39+ Treg cells, a subset of CD4+T cells; conventional CD4+ T cells, or CD8^+^ T lymphocytes increased the expression of immunosuppressive molecules, such as TGF-β, IL-10, COX-2, CD39, and CD73 [41]. The role of TD-EVs carrying miRNA in immunosuppression has been described in a few studies. For instance, overexpression of five miRNAs was reported in EVs derived from nasopharyngeal carcinoma cells. These overexpressed miRNAs reduced the MAPK signaling in T cells, leading to impaired T cell proliferation and differentiation [42]. Moreover, miRNA from TD-EVs also regulates the activity of other immune cells, such as NK cells, B cell monocytes, and DCs ( reviewed by Michael W Graner) [43]. TD-EVs also regulate the function of mesenchymal stem cells (MSCs), which support cancer progression by creating an immunosuppressive microenvironment. For instance, heat shock protein (Hsp)70 on the surface of EVs from lung tumor cells activated NF-κB signaling and elevated the secretion of proinflammatory cytokines by MSCs, thus promoting tumor growth [44]. Furthermore, TD-EVs carrying enzymatically active ectonucleotidases CD39 and CD73 suppress the activation of T cells and B cells. CD39 and CD73 secrete an immunosuppressive factor, adenosine, and negatively regulate the immune response [41,45].
**(c)**  Effects on differentiation of immune cells: EVs derived from breast cancer cells increased the TGFβ-mediated phosphorylation of Smad2/3 and STAT3 in T cells, thereby changing the phenotype to Treg cells [46]. TGFβ is one of the major immunosuppressive cytokines present on the surface of EVs. TD-EVs-associated TGFβ1 suppressed the activity of NK cells by lowering the NKG2D expression in AML patients and suppressed T cell proliferation in breast cancer [46,47,48]. EVs derived from human multiple myeloma cells, renal cells, and murine breast carcinomas triggered the differentiation and proliferation pathways in MDSCs, which depends on the activation of STAT3 signaling and also the presence of prostaglandin E2 PGE2, Hsp72, and TGF-β in the TD-EVs cargo [7,49,50]. Furthermore, EVs derived from ovarian, pancreatic, and colon cancers shift cancer-suppressive M1 macrophage to a tumor-supportive M2 phenotype [51,52]. Overall, these findings support the immunosuppressive ability of TD-EVs that negatively regulate the function of immune cells by transferring bioactive molecules, such as nucleic acids and/or proteins.

### 3.2. Tumor-Derived Extracellular Vesicles Stimulate the Immune Response

In addition to the immunosuppressive molecule, TD-EVs also carry costimulatory molecules, such as MHC class I and class II, growth-promoting cytokines, and some TAA. However, it remains unclear how TD-EVs deliver multiple signals to regulate dual response in immune cells. It is believed that these inhibitory or stimulatory signals are delivered simultaneously. Nonetheless, the nature and type of recipient cells and prevailing conditions in TME may play a role in reprogramming the immune response rather than the cargo of inhibitory or stimulatory signals in TD-EVs. This contradiction in immune-cell response might be due to the possibility of recipient cell-mediated crosstalk between the host immune system and tumor. Several studies have shown that TD-EVS can increase the differentiation and antigen-processing properties of DC in TME, suggesting that TD-EVs may enhance the efficacy of antitumor vaccines. To support this fact, recent in vivo studies have shown that incorporation of TD-EVs into anticancer vaccines induce immunostimulatory effects [53]. DCs loaded with EVs derived from glioma activated the tumor-specific T-cell response in vivo. Treatment with these EVs stimulated the upregulation of costimulatory receptors CD80, CD86, and MHC II molecules on DCs. The vaccination efficiency of DCs loaded with TD-EVS was high in mice compared to DCs loaded with tumor cell lysates [54,55]. Besides, EVs loaded with α-galactosylceramide and tumor peptide antigen led to the activation of NK and T cells [56]. Similarly, the antitumor immune response was generated from EV with HSPs derived from heat-shocked lymphoma cells [57]. Studies have shown that DCs loaded with TAA also produce EVs that transfer MHC complex to other APCs or immune cells. Such DC-derived EVs could activate naïve CD4^+^ T cells in vivo and are being employed as cancer vaccines [12,58]. 

M1 macrophages may mediate the immunostimulatory effects of TD-EVs in TME, which increases the release of cytokines, such as IL-6, IFN-γ, TNF-α, and IL-12, thus promoting the T cell-mediated immune response. For example, EVs derived from melanoma, gastric cancer, and breast cancer cells uptake by macrophages that stimulate the NF-κB pathway and increase the expression of proinflammatory factors [59,60,61]. This is the best example of juxtracrine effects mediated by TD-EVs by reprogramming DCs and M1 macrophages in TME to enhance antitumor immune responses. Considerable efforts are being made in this direction to enlist TD-EVs as a potential tool for antitumor vaccines but require additional in vivo studies to select immune-potentiating EVs. These immune-potentiating TD-EVs must be enriched in costimulatory molecules, such as cell surface receptors/ligands, for efficient uptake by APCs. The lumen of these EVs must carry mRNA/miRNA that redirects recipient cells to generate cytokines promoting immune cell response. This can be achieved by ex vivo modification of EVs, which is currently a part of exosome research [62].

## 4. Immune Cells Derived Extracellular Vesicles

### 4.1. B Cell-Derived Extracellular Vesicles

EVs derived from B cells can generate an immune response via multiple processes. They carry B cell receptor, MHC-I, MHC-II proteins, costimulatory molecules, human leukocyte antigen, and activate antigen-specific T cells and induce immune responses [23,63]. They also express a high level of cell surface and adhesion/integral membrane proteins; ICAM-1 (CD54) and integrin that help in mediating cell–cell adhesion through the extracellular matrix and aid in target cell delivery [21]. B cell-derived EVs carrying a different type of antigens may elicit different types of immune response. 

### 4.2. DC-Derived Extracellular Vesicles

Dendritic cells are one of the most potent APCs and can produce a huge number of MHC-II molecules through the DC-derived extracellular vesicles (DC-EVs) pathway. DC-EVS has been shown to activate antigen-specific T cell-mediated cytotoxicity [24]. T cell receptor complexes are engaged in the activation of DC-EVS-mediated T cell activation while adhesion molecules facilitate the delivery of exosomal cargoes to targeted cells as in the case of B cell-derived EVs [64].

DCs can present the antigenic peptides from exogenous proteins that are picked up by the cells through endocytosis. Given the importance of DCs in regulating immune response, most of the studies on DC-EVS are focused on immunotherapy in cancer treatment that has been translated into clinical benefits [65,66,67]. Moreover, EVs derived from dendritic cells also play an important role in other diseases, such as autoimmune diseases and cardiovascular diseases [68,69]. The molecular composition of DCs and their EVs are similar, including T cell costimulatory molecules, MHC-peptide complexes, and other cellular proteins that interact with immune cells [70].

### 4.3. T Cell-Derived Extracellular Vesicles

Like any other APCs, T cells also produce EVs with multiple biological and physical characteristics. EVs derived from T cells are known to exert similar functions as that of their parent T cells. Different T cell subsets have been identified; each plays an important role in cell-mediated and humoral immunity [71]. T cell-derived EVs express a large number of surface proteins, such as glucocorticoid-induced tumor necrosis factor receptor, MHC I/II complex lymphocyte function-associated antigen 1/2, tumor susceptibility gene 101, FasL or CD95L, chemokine receptor type 4, or CD184 and TCR [72]. Recently, Fu at al. demonstrated that EVs derived from CAR-T cells carry CAR on their surface that express a high level of cytotoxic molecules, granzyme and perforin, that inhibit tumor growth. Moreover, compared to CAR-T cells, CAR EVs do not express the programmed cell death protein 1 (PD1), and their antitumor effects cannot be weakened by recombinant PD-L1 treatment [73].

### 4.4. NK Cell-Derived Extracellular Vesicles

NK cells are a type of granular lymphocytes and a component of innate and adaptive immunity. They can kill cancerous cells and pathogen-infected cells and stimulate the adaptive immune response by secreting chemokines and proinflammatory cytokines [74]. Activated NK cells produce EVs that express cytotoxic protein viz. granulysin, perforin, FasL, and granzymes A and B, which is involved in caspase-mediated tumor cell lysis [75]. Similarly, FasL, perforin, and TNF-α secreted from human NK92 cell-derived EVs mediated lysis of melanoma cells in vitro and in vivo [76]. Recently, an increase in the secretion of EVs has been observed when NK cells were previously exposed to neuroblastoma cells. These NK cells derived EVs displayed a greater cytotoxic effect against neuroblastoma tumor than EVs derived from naïve NK cells, suggesting that EVs derived from activated NK cells could be used as an immunotherapeutic in disease treatment [77]. 

## 5. Extracellular Vesicles in Tumor Microenvironment Remodeling

Premetastatic niche formation is the chief event that confers the ability of colonization to tumor cells to distant locations for further metastasis. Previous studies have reported the involvement of EVs with cytokines and other mediators in the establishment of the TME to form a premetastatic niche. Tumorigenic EVs are known to transfer cellular oncogenic cargo to either cancerous or normal cells, thereby modulating the gene expression pattern of the recipient cells, leading to cancer survival, metastasis, and drug resistance [78]. EVs are also the major role player in the remodeling of the extracellular matrix, angiogenesis, and thrombogenesis [79]. Recently, an intercellular communication study performed with metastatic brain tumor glioblastoma demonstrated the involvement of EVs in tumor angiogenesis, neovascularization, and hypoxia-dependent inter-tumor communication during cancer progression [79]. Gastric cancer-derived EVs activate the tumor-associated macrophages, which are considered to have an M2 macrophage-polarized phenotype. These activated macrophages secrete vascular endothelial growth factor(VEGF,) IL6, miRNAs, and transcription factors, which together promote angiogenesis in many cancers. TD-EVs contain TGF-β, which triggers the differentiation of fibroblast cells to cancer-associated fibroblasts and promotes extracellular matrix remodeling and angiogenesis by releasing matrix metalloproteinase and cytokines [80,81]. During hypoxia, EVs derived from cancer cells are enriched in angiogenic factors, such as VEGF and Hypoxia-inducible factor 1-alpha having metastatic potential; this suggests that tumor cells can adjust to a hypoxic microenvironment by secreting EVs to promote angiogenesis or metastasis [82]. Additionally, in high-grade ovarian cancer, EVs carrying VEGF contribute to the crosstalk between cancer and endothelial cells for angiogenesis and metastasis [83,84,85]. Hence, EVs seem to be an important driver for tumor metastasis and development (Figure 1).

## 6. Extracellular Vesicles in Transcriptional Regulation

EVs derived from tumors carry a cargo of transcriptional factors, such as mRNA, miRNA, or proteins that may affect cellular transcription, thus altering the signaling events in normal and cancer cells that may have direct impacts on gene expression and protein synthesis in target cells. In view of this, many studies have demonstrated a linear relationship between the association of EVs and transcriptional regulation [86]. For example, in the nutrient-deprived tumor microenvironment, the upregulation of EGFR, VEGF, and HIG1A in nearby cells can be induced by EVs [87]. Indeed, proteomic data from human medulloblastoma cell lines and murine brain tumor cell lines revealed the number of proteins, such as ribonuclear proteins and various transcription factors. Few were found to be involved in transcriptional and translational regulation with a putative effect on downstream signaling pathways in cancer [87]. Moreover, these transcription factors also regulate the cellular function of immune cells and any alteration in immune cell functionality due to transcriptional dysregulation may inhibit the ability of the immune system to limit tumor progression.

## 7. Tumor-Derived Extracellular Vesicles Mediate Resistance to Immunotherapy

Immunotherapy was a breakthrough in the history of cancer therapy. However, patients treated with immunotherapy have shown varying response rates among cancers within the same malignancy cohorts. These variations may be due to the specificity involved in eliciting an immune response, which overcomes the mechanisms employed by cancer cells to evade immune surveillance and ensure that the activated immune cells have access to the malignant tissue. Increasing evidence confirmed that TD-EVs carrying immunosuppressive biomolecules inhibit the antitumor function of immune cells and interfere with existing immunotherapies [47]. For example, TD-EVs carrying TAA decrease the efficacy of tumor-targeting antibodies. These TD-EVs are abundantly expressed in the body fluid of cancer patients, and therapeutic antibodies can be absorbed by TAA present on EVs, blocking the access of antibodies to tumor cells, and thus suppressing their antitumor effects [80,81]. Similarly, there are several mechanisms by which EVs carrying PD-L1 mediates resistance to immune checkpoint inhibitor therapy and promotes tumor growth. For example, TD-EVs increase the expression of PD-L1 and the release of cytokines by myeloid cells to inhibit the function of T cells. Secondly, exosomal PD-L1 is bound to anti-PD-L1 antibody and mediates resistance to anti-PD-1/PD-L1 immune checkpoint therapy and blocks the activation of T cells, thereby promoting tumor growth [29,38,88]. Additionally, EVs carrying inhibitory ligands, such FasL, interfere with the Fas/Fas-ligand pathway and induce apoptosis of activated T cells following cancer vaccines or adoptive T or NK cell therapy [89,90,91]. These results conclude that TD-EVs can interfere with immune cells used for adoptive cell transfer and immune checkpoint inhibitor therapy. However, the precise mechanism of resistance to immunotherapy by TD-EVs is largely unclear. 

## 8. Extracellular Vesicles as a Carrier of Cancer Therapy

**Figure 2:**
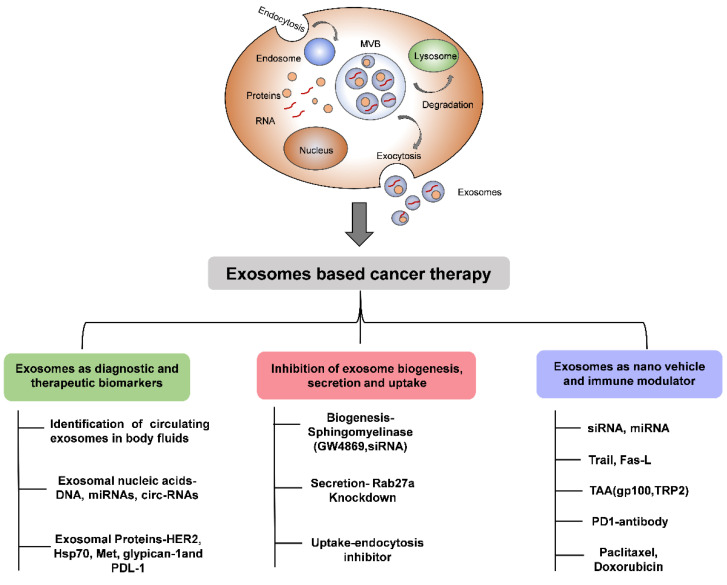
Schematic diagram showing the importance of EVs in cancer therapy.

Numerous strategies have been employed to increase the immunostimulatory effects of EVs. For example, loading the EVs with immunotherapy elements, such as TAA (gp100 and TRP2) and adjuvants, has shown the induction of strong antigen-specific antitumor immune response [92]. In a study by Morishita et al., loading of murine melanoma B16-BL6 cell-derived EVs by TAA and streptavidin-lactadherin enhanced the delivery of biotinylated CpG DNA and immunization of B16-BL6 tumor-bearing mice with these engineered EVs resulted in strong antitumor effects [93]. Similarly, EVs secreted by MDA MB-231 cells were modified to express staphylococcal enterotoxin B. This facilitated the activation of T cells and treatment with these EVs induced significant apoptosis in MDA MB-231 breast cancer cells [94]. Furthermore, the upregulation of Let-7i and miR-142 in TD-EVs increased the maturation of DC and cytokine release [95]. Similarly, EVs engineered to express PD-1 membrane receptors could enhance the antitumor response by disrupting the PD-1/PD-L1 immune inhibitory axis [88]. Yuyang Zhang et al. showed that the transfer of exosomal miR-124 secreted by ovarian cancer cells inhibited the sphingosine kinase 1 (SPHK1) expression in cancer-associated fibroblasts and reduced tumor growth. The expression of SPHK1 is increased in various cancers, including breast cancer, gastric cancer, colon cancer, glioblastoma tissues, and cells, facilitating tumor growth. Thus, targeting SPHK1 by exosomal miR-124 may be of clinical use in cancer treatment [96]. Recently, Zhang et al. showed that exosomal transfer of long non-coding RNA contributed to the malignant phenotypes in hepatocellular carcinoma cells, especially those in residual tumors after insufficient radiofrequency ablation [97]. In addition to this, the loading of B cell-derived EVs with miR-155 induced the differentiation and activation of macrophages to a tumor-inhibiting M1-phenotype [98]. It has been reported that TD-EVs express HSPs, such as Hsp70 and Hsp90, on their surface, which activates DCs, and EVs from these activated DCs loaded with tumor-associated chaperons could elicit a strong T cell immune response in mice with intracranial glioma, suggesting the immunostimulatory effect of TD-EVs loaded with chaperons [99]. Moreover, surface proteins present on DC-EVs, such as ICAM-1, MFG-E8, and tetraspanin, facilitate their interaction with target tumor cells. Data from preclinical studies have shown the activation of CD4^+^T cells and CD8^+^ T cells by DC-EVs in non-small cell lung cancer and melanoma patients with the potential to maintain the feasibility and safety of the application [78]. The use of DC-EVS and TD-EVs in anticancer therapy is worth considering because of their efficacy and safety, which is supported by ongoing research with promising data on using DC-EVS vaccination, but the therapeutic success and activation of the immune response are limited [66]. Another possible therapeutic strategy is blocking the biogenesis and secretion of EVs [100]. Targeting neutral sphingomyelinase using siRNA and drug molecules inhibits the synthesis of ceramide, a key molecule in the biogenesis of EVs [101]. Various Rab proteins are associated with the selective packaging and release of EVs in both normal and tumor cells [100]. Targeting Rab27a in highly metastatic melanoma and breast cancer cells significantly reduced the tumor growth and metastasis [4,100,102]. In addition to inhibiting EVs biogenesis, targeting their specific transmembrane integrins and oncogenic cargo (i.e., MIF, MET, KIT) may decrease tumor growth and metastasis. EVs can be employed as promising nano vehicles for the targeted delivery of therapeutic RNA, protein, and chemotherapeutic drugs. EVs carrying chemotherapeutic drugs, such as methotrexate and curcumin, have shown promising anticancer effects in various cancers [103]. Similarly, EVs loaded with siRNA targeting KRAS have significantly suppressed the progression of pancreatic tumors and increased overall survival in mouse models [104]. EVs carrying siRNA against Bace1 and MAPK genes efficiently reduced the expression of these genes in neurons, monocytes, and lymphocytes [105,106]. In summary, engineering the EVs, antagonizing their synthesis, release, and uptake, may benefit cancer therapy (Figure 2).

## 9. Extracellular Vesicles as a Biomarker in Cancer

The fact that liquid biopsy has gained much attention of the scientific community and clinicians cannot be neglected. Liquid biopsy offers a more convenient diagnostic approach where surgical removal of tissue samples is not feasible. Furthermore, it also provides researchers with access to a non-invasive diagnostic approach. Analysis of EVs in liquid biopsy is also not an exception in the early diagnosis of cancer and associated malignancies. Compared to ctDNA and cell-free tumor RNA, EVs are highly stable in blood plasma and body fluids. Ultracentrifugation is the most common and traditionally accepted method for exosome purification from cell culture supernatants or blood plasma. Apart from ultracentrifugation, exosome precipitation using a commercially available kit, such as Exoquick, Invitrogen Total Exosome Isolation reagent is used for research purposes. However, these kits are not suitable when it comes to clinical use for diagnosis or treatment. Previously, several studies have described different methods of isolation and characterization of EVs and their specific markers. The standardization of sample collection, isolation, and analysis methods for exosome isolation from small amounts of biofluids, such as blood plasma, has been published in several previous International Society for EVs position papers [107,108]. Circulating and TD-EVs have enormous macromolecules, such as circulating tumor DNA, proteins, and aberrant miRNAs, that can be explored as tumor determinants and later on can be analyzed as tumor biomarkers [109]. For sure, EVs carrying glypican-1 and PD-L1 serve as one of such non-invasive diagnostic biomarker in early detection of pancreatic cancer and melanoma [110]. EVs from melanoma patients consist of the melanoma-specific protein, very late antigen, tyrosinase-related protein-2, MET, caveolin-1, and Hsp70 as compared to the healthy control, rendering them a potential biomarker in melanoma [102]. Similarly, the miRNA monograms of TD-EVs also serve as potential diagnostic biomarkers in a variety of cancers, including glioblastoma, ovarian cancer, colon cancer, colorectal cancer, and prostate cancer [111,112]. Apart from miRNAs, circular RNAs, which are more prevalent in EVs derived from cancer cells and serum samples of cancer patients, may serve as unique EV-based cancer biomarkers [113]. 

## 10. Conclusions and Future Perspectives

In the past 10 years, EVs have played a diverse role in immune regulation in the tumor microenvironment. Numerous studies have provided evidence supporting their potential in immunotherapeutic strategies in various clinical conditions, including cancer. TD-EVs are of special interest as they carry and transfer signals that are either stimulatory or inhibitory depending upon the nature of targeted cells and cellular composition of the tumor microenvironment. Nonetheless, there is still a long way to go to fully understand the molecular mechanism of exosome-mediated transcriptional or translational changes and immune regulation in cancer development for a comprehensive antitumor regimen. Due to the complexity in the nature and the function of EVs, further improvement in several strategies is required, for example, better methods for isolating tumor EVs are needed. So far, ultracentrifugation is a widely used technique for EV isolation, which is time-consuming and compromises with the purity of EVs. Furthermore, a better understanding of the packaging of EVs in cancer cells is required, which eventually will help in early diagnosis and treatment prediction of cancer. In addition to this, the engineering of EVs will enhance their efficacy in delivering drugs, antigens, or nucleic acids to the targeted cells. Moreover, more clinical trials should be done in different types of cancers to validate the use of EVs in cancer diagnosis and therapy.

## Abbreviations

TD-EVs	Tumor-derived extracellular vesicles
TAA	Tumor-associated antigens
DC	Dendritic cell
CTL	Cytotoxic T lymphocytes
APC	Antigen-presenting cell
EVs	Extracellular vesicles
SPHK1	Sphingosine kinase 1
FasL	Fas ligand
DC-EVs	DC-derived extracellular vesicles
MSC	Mesenchymal stem cells
MHC	Major histocompatibility complex
MDSC	Myeloid-derived suppression cell
NK	Natural killer cells
TNF-α	Tumor necrosis factor alpha
TRAIL	TNF-related apoptosis-inducing ligand
TGF-β	Tumor growth factor beta
VEGF	Vascular endothelial growth factor
